# The subjective value of a smile alters social behaviour

**DOI:** 10.1371/journal.pone.0225284

**Published:** 2019-12-02

**Authors:** Erin A. Heerey, Thandiwe S. E. Gilder

**Affiliations:** 1 Psychology Department, University of Western Ontario, London, Ontario, Canada; 2 School of Psychology, Bangor University, Bangor, Wales, United Kingdom; Univdersity Hospital of TübingenUniversitatsklinikum Tubingen, GERMANY

## Abstract

Face-to-face social behaviour is difficult to explain, leading some researchers to call it the “dark matter” of psychology/neuroscience [1]. We apply an idea from neuroeconomics to this problem, suggesting that how people subjectively value facial expressions should predict usage differences during unconstrained interaction. Specifically, we ask whether the subjective value of smiles is malleable as a consequence of immediate social experience and how this relates to smiling during face-to-face interactions. We measured the value of a smile in monetary terms and found that increases in people’s social neediness caused devaluation of polite smiles but no changes in how they valued genuine smiles. This result predicts that participants induced to feel high levels of social need should be less responsive to their social partners’ polite smiles in a subsequent unconstrained social interaction. As expected, high social-need participants returned fewer polite smiles when interacting with a partner, leading to poor interaction outcomes. Genuine smile reciprocity remained unchanged. Findings show that social states influence real-world interactions by changing social-cue valuation, highlighting a potential mechanism for understanding the moment-to-moment control of social behaviour and how behaviour changes based on people’s subjective evaluations of the social environment.

## Introduction

Genuine smiles of pleasure are rewarding social cues that cause positive feelings in receivers and lead people to anticipate positive social outcomes [2–4]. When provided as reinforcers for social decisions, genuine smiles enhance learning and activate reward circuitry in the brain [5, 6]. Polite smiles occur in the absence of positive emotion, when politeness rules necessitate a smile [7]. Although not typically considered rewarding [3, 8], polite smiles are nonetheless important social tokens [9]. Indeed, both genuine and polite smiles are highly likely to be reciprocated in face-to-face social interactions and good smile reciprocity enhances liking for a social partner [10–12]. Thus, these smiles likely play an important role in social decision-making.

Examined in the context of economic choices, smiles are also important social cues. Findings suggest that smiles alter decision-making such that participants are willing to invest more money with smiling versus non-smiling partners in trust games, even when returns are lower [13, 14]. Smiles also add value to choices in simple decision-making tasks, meaning that participants are willing to forego the chance to earn monetary rewards in favour of the chance to see genuine smiles [15]. Thus, like financial incentives, smiles may shape the decisions people make by altering the “utility,” or subjective value, of the actions with which they are associated. If fluctuations in smile value change moment-to-moment responses to these cues in social interactions, such a result would highlight one mechanism underpinning real-world social behaviour. Here we ask whether the subjective value of a smile depends on social states, just as physical states (e.g., hunger or satiety) change the subjective value of associated stimuli (e.g., food [16, 17]). We then ask whether alterations in smile value determine responses to smiles in face-to-face interaction. An important dimension of people’s social states is the degree to which they feel accepted versus rejected by their peers [18–21]. The experience of social rejection, leading to a state of enhanced social need, dramatically influences immediate emotions and social behaviours [22–26] and, in cases of chronic rejection, predicts poor long-term outcomes [27, 28]. We anticipate that altering social need state by inducing immediate feelings of social rejection will influence the subjective values of different types of smiles. For example, after experiencing a social rejection manipulation, individuals show better ability to discriminate genuine smiles from polite smiles, greater visual attention to genuine smiles, and express greater desire to work with genuinely versus politely smiling partners [29–31].

Such findings have led researchers to hypothesize that genuine smiles become more valuable under states of high social need because they predict positive social outcomes, thereby appeasing rejected individuals’ need for social belonging [18, 29, 32, 33]. However, whether smile-value is indeed malleable remains unknown, as this has never been directly tested. Moreover, because genuine and polite smiles are typically compared to each other, rather than to a neutral/control condition (e.g., [29, 34]), it remains unclear whether rejection-induced changes in social state alter the value of genuine smiles, polite smiles, or both. This is an important question because it speaks directly to a possible mechanism by which social state changes may alter face-to-face social behaviour and subsequent social outcomes after enduring a social rejection [18, 19, 21, 22, 35, 36].

If rejection experiences cause changes in smile valuation that in turn affect social behaviour, these changes are likely to have major downstream consequences for interaction. For example, previous research shows that in natural interactions participants reciprocate 70% to 80% of their partners’ smiles. More importantly however, they match their partners’ smile types such that a partner’s genuine smiles lead people to return genuine smiles and polite smiles lead to polite smile returns [10, 37]. Evidence additionally suggests that the value of a smile shapes some aspects of this reciprocity, such as its timing [10]. Thus, changes in smile value as a result of social state fluctuations may change how participants view and experience partners’ social behaviour.

We note that the purpose of this research is not to make a direct contribution to the social rejection literature. Rather, the major point of this work is to contribute to the literature on how changes in people’s subjective affective experiences drive their face-to-face social behaviour. We consequently propose the hypothesis that social state changes will alter the subjective utility of smiles, and thereby change how people respond to their partners’ smiles. This work therefore offers the first direct test of the longstanding prediction that rejection-induced changes in social need enhance the value of genuine smiles and we include the critical neutral condition, allowing us to examine smile-value changes for genuine and polite smiles separately.

To investigate the effects of changes in how people respond to smiles, we adopt a neuroeconomic approach to the problem of quantifying smile utility. Specifically, we adapted a task commonly used in studies of economic utility (e.g., [38, 39, 40]) that allows us to measure the value of genuine and polite smiles in monetary terms [15, 41]. In utility tasks, participants must choose between pairs of stimuli that vary in value on one or more dimensions. One of the dimensions is often “subjective,” for example, how much a particular person desires merchandise from a set of companies (e.g., Apple, Samsung). The other dimension is typically quantifiable using a standard currency, for example, how much the person is willing to pay to obtain the branded merchandise (e.g., [42, 43]).

In order to represent subjective value in monetary terms, participants make choices amongst pairs of stimuli. Because all possible pairings of stimuli are presented and participants must always select one stimulus per trial, their selections across this decision-space provide a measure of their relative preferences for each stimulus in the set. It is possible to precisely quantify subjective preferences by determining how much additional currency people are willing to pay in order to receive a desired, relative to a less desired stimulus. This method is advantageous to simple preference reporting, because it enables precise quantification of relative preferences for one stimulus dimension (e.g., brand-name) standardized in terms of the other dimension (e.g., cost).

Our version of this task uses a set of faces that provide monetary reinforcement with either higher or lower frequency and give different types of social feedback believed to vary in subjective value [38, 40]. We use a two-step procedure in which participants experience and learn both the monetary and social values of our stimuli, followed by a test phase in which they must choose between stimuli presented in pairs. Based on the existing literature, we predict that rejection-induced increases in social need will enhance the subjective value of genuine smiles. That is, participants who experience a social rejection should be willing to forgo a greater amount of money for the chance to see genuine smiles relative to participants who have not undergone a social need induction.

## Experiment 1

We begin by quantifying how social state (e.g., feeling rejected versus accepted by a future social partner) influences the subjective value of smiles.

## Methods

### Participants

Ninety psychology undergraduates (62 female; mean age: 20.47 years, SD: 4.56) participated in “a study of personality characteristics and first impressions” in exchange for partial course credit and a small performance-based monetary bonus. We determined the sample size before the start of data collection, based on a power analysis. We estimated effect sizes based on effect-size data reported in two previously published experiments examining responses to genuine and non-genuine smiles [29, 34]. A G*Power [44] analysis (effect size f = .591, *α* = .05, 1-*β* = .95) suggested groups of 16 participants each. However, we oversampled to obtain 30 participants per condition (after exclusions). Potential participants were pre-screened to rule out the presence of social anxiety (defined as a score >60 on the Interaction Anxiousness Scale, [45]), which may interfere with the processing of social stimuli [46]. No person scoring more than 60 on the Interaction Anxiousness Scale was invited to participate in the present study. Participants provided written consent and the Bangor University School of Psychology Ethics Committee approved the experiment and all study procedures (Likewise for Experiment 2 below). We excluded data from four additional participants because they reported suspicion about the experiment’s manipulation.

### Procedure

Participants arrived for the experiment in pairs to give the impression that they would be partners for a social interaction. In reality, they did not have a face-to-face interaction but instead completed a computer task measuring the utility of genuine and polite smiles, relative to neutral faces. Upon arrival, the experimenter seated participants at computers in separate rooms where they completed a series of questionnaires measuring aspects of personality (not analyzed), along with the Positive and Negative Affect Schedule (PANAS [47]) to measure mood. We measured social need state by inserting six rejection-related words into the PANAS at random points (slighted, misunderstood, rejected, liked [reversed], respected [reversed], supported [reversed]; Cronbach’s *α* = .707). After completing these measures, participants viewed a personality profile, ostensibly of the partner. In reality, however, all participants viewed the same profile.

Based upon this personality profile, participants responded to a series of statements about how much they looked forward to meeting the partner (e.g., “I’m looking forward to meeting my partner”) and then received false feedback about how much the partner looked forward to meeting them (for similar procedures see [48, 49]). The computer then randomly assigned participants to one of three feedback conditions (see Supporting Information Pilot Results and S1 Fig for full text and validation information). These were 1) acceptance feedback (low social need; “Your partner is looking forward to meeting you.”; n = 30); 2) social rejection feedback (high social need plus high negative affect; “Your partner is not looking forward to meeting you.”; n = 30); and 3) negative, non-rejection feedback (high negative affect only; “Your partner is not looking forward to meeting anyone.”; n = 30). Importantly, this third condition allowed us to determine whether changes in smile utility related specifically to social need state versus negative emotion more generally. An independent set of pilot participants assessed the manipulation to ensure that it was both believable and caused the desired outcomes in receivers (see S1 Fig for pilot results). Finally, because the computer assigned feedback to each participant, the experimenter was blind to participant condition until the conclusion of the experiment.

After viewing the feedback, participants completed a second PANAS (including social-need items), a computer-based smile-valuation task that allowed us to measure the utility of smiles in monetary terms (see below), and a short task in which they distinguished genuine smiles from polite smiles. Following the tasks, the experimenter probed participants for suspicion about the manipulation, explained the manipulation and fully debriefed them, paid them based on their earnings in the smile-valuation task and finally gave them the opportunity to provide fully informed consent using a secret “sealed envelope” procedure, which ensured that the experimenter was unaware of their final response until after participants had been dismissed. No participant declined consent.

### Smile-valuation task

This task has both exposure and test phases. The purpose of the exposure phase was to allow participants to become familiar with a set of faces that varied on two reward dimensions (monetary value and social value). This phase allowed us to ensure that participants experienced the exact same reinforcement frequencies and types in each monetary and social value condition. During this phase, participants experienced a set of six opponents, each identified by a photograph of a face.

**Fig 1:**
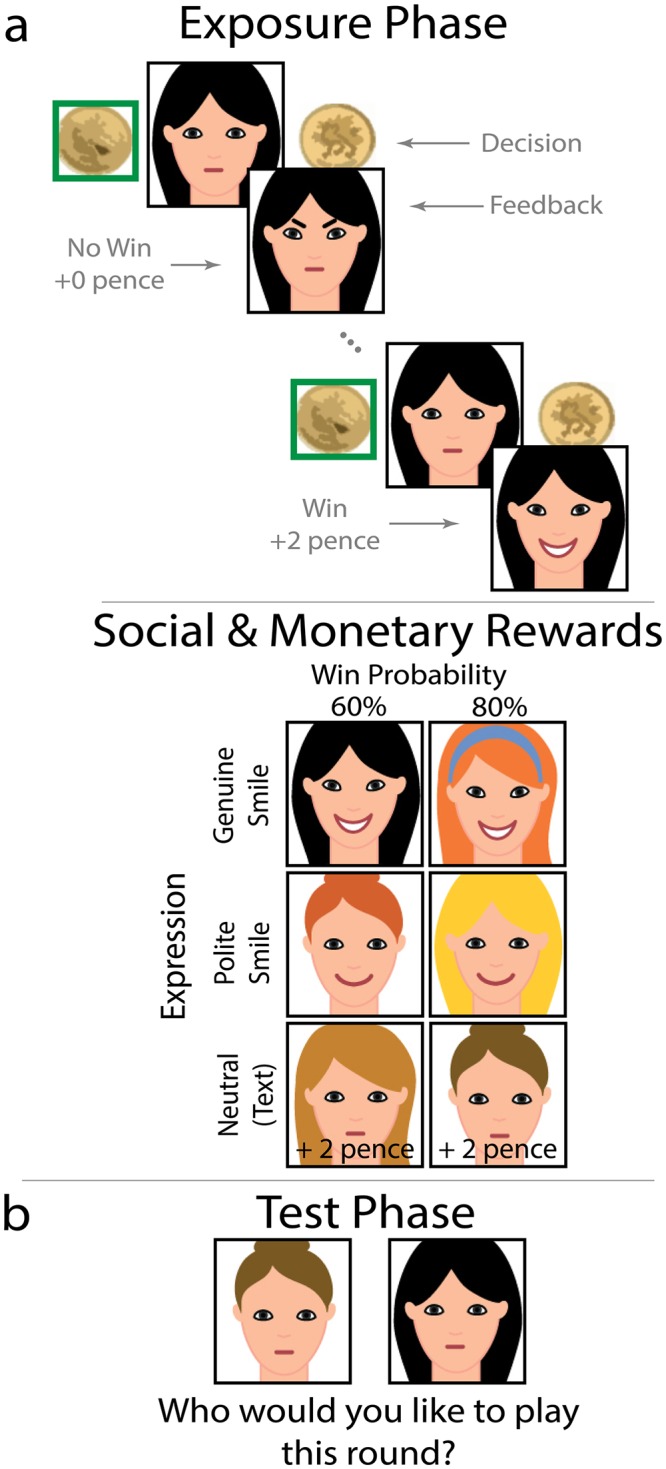
Smile valuation task. In the exposure phase, **a)** Participants attempted to choose the same side of the coin as a computerized player (indicated by a photograph) using a key press. Players indicated match (worth .02GBP) and non-match outcomes (worth 0GBP) with either facial or text displays. There were three high-value faces (providing win feedback on 80% of trials, regardless of participants’ choices) and three low-value faces (60% win feedback). One high-value and one low-value face provided match feedback with genuine smiles; likewise for polite smiles and text feedback. **b)** On each trial in the test phase, participants chose the “most valuable” player from amongst pairs of players, prior to playing out the round. All possible pairings were represented. These choices served as the dependent variable in the task. This task is identical to the task described in [41]. Note that the images participants viewed during that task were photographs of faces, rather than the schematic faces illustrated in the Fig.

On each trial of the exposure phase, participants viewed one opponent, neutrally posed, in the center of the screen and attempted to win money by choosing the same side of a virtual coin as the opponent (Fig 1a). To indicate a match (each worth .02GBP), the opponent smiled genuinely (involving zygomaticus major and orbicularis oculi); smiled politely (zygomaticus major only); or remained in the neutral pose with text superimposed on the screen indicating the win (for a full description of the smile stimuli and their psychometric properties, see [41]). On non-win trials, superimposed text appeared indicating a non-win for the neutral opponents whereas the other opponents frowned.

Unbeknownst to participants, match feedback did not depend on which side of the coin they chose. Instead, three opponents (randomly determined) provided match feedback on 80% of trials. The remaining opponents provided match feedback on 60% of trials. Two opponents (one 80% and one 60%) always displayed genuine smiles on match trials, two displayed polite smiles (one 80% and one 60%) and the others remained neutral (Fig 1). These reward values remained the same across exposure and test phases. Participants experienced three blocks of 60 exposure-trials, viewing each opponent 10 times per block in random order.

The test phase allowed us to determine the degree to which participants valued genuine and polite smile feedback in monetary terms. When given a choice, between a pair of opponents, the degree to which a participant is willing to forgo the chance to win money in favour of the chance to see each smile indicates that smile’s subjective value to the participant. On each trial, participants chose the “most valuable” opponent from amongst a pair of neutrally posed opponents (Fig 1b). After participants chose an opponent, trials continued as in the exposure phase. All fifteen possible opponent pairings appeared eight times each, in random order (120 test-trials), with each face in a pair appearing as often on the left as on the right in the pairing.

Participants’ choices served as our measure of their preferences for social, relative to monetary value in this experiment. That is, if participants genuinely prefer one face to another in a given pairing, they will choose that one more often. If they are indifferent with respect to which face they prefer in the pairing, they will choose each face in that pairing with similar frequency. For example, if a participant strongly prefers smiling to neutral faces, that participant will select smiling faces whenever they are asked to choose between a face that smiles and a face that does not. This will be true even if the smiling face has a lower probability of giving a monetary reward. Based on the relative differences between the monetary and social values of the faces in each pairing, and participants’ preferences for one face versus another, it is possible to estimate how much a participant values smiles to neutral faces, expressed in monetary terms.

To ensure that specific opponent/value pairings did not affect results, each opponent’s face appeared in each monetary/social value combination with approximately equal frequency across participants. Half the participants saw male opponents and half saw females, counterbalanced by participant gender. The experiment was programmed using the Psychophysics Toolbox extensions [50] for MATLAB (The Mathworks).

### Smile discrimination task

Participants additionally completed a smile discrimination task to ensure that any differences in choice behaviour did not relate to the ability to discriminate genuine from polite smiles. Participants viewed a series of 24 still images of faces (12 female) displaying either genuine or polite smiles and indicated with a key-press which type of smile the face displayed.

### Data analysis

Because participants experienced all opponent pairings, it was possible to determine how different opponent features changed participants’ selections based on differences in the features of the selected and unselected opponents within a given pairing. We individually estimated the degree to which money (lower versus higher value) and smiles (genuine versus neutral; polite versus neutral) influenced participants’ decisions in the test phase of the smile-valuation task by applying Utility Theory [51] to the choice data (see also [15, 41]. This analysis allowed us to estimate the subjective value of the differences between opponents’ social and monetary values in a given opponent-pairing by quantifying how these differences shaped participants’ choice behaviour. Each participant’s raw choice data were individually fit using the logistic response function:

$${\text{P}}_{Opponent\;A}=\text{exp}\left(\theta \right)/1+\text{exp}\left(\theta \right)$$ (1)

where *P*_*Opponent A*_ is the probability of choosing the left over the right opponent in an opponent pair (see Fig 1b), and *θ* is the difference in opponents’ utilities, modelled by the linear function:

$$\theta =\beta_{Money}X_{Money}+\beta_{Genuine}X_{Genuine}+\beta_{Polite}X_{Polite}$$ (2)

In this equation, *X*_*Money*_ codes the difference between the left and right opponents’ expected monetary values. A stimulus’s expected value is its win value multiplied by its win probability [52]; in this case, .02GBP multiplied by either an 80% or a 60% chance of winning. Thus, *X*_*Money*_ received a value of .4 if the left opponent was better than the right, -.4 if the right opponent was better, and 0 if they were equal. *X*_*Genuine*_ coded genuine smiles. If the left opponent smiled genuinely and the right was neutral, we coded this as 1, if expression types were reversed, we coded this as -1, and if both or neither opponent smiled genuinely, this was coded 0. *X*_*Polite*_ was coded in similar fashion for polite smiles. The *β*s are the unstandardized logistic regression weightings for each model component. These were estimated using a robust, iteratively re-weighted least squares algorithm to obtain the maximum likelihood estimates for each term in the model [53]. The regression coefficients from this model were examined statistically using MANOVA with feedback type (acceptance, negative non-rejection, rejection) as the independent variable.

Our smile discrimination task data were analyzed using a signal detection framework. We recorded a hit when participants correctly identified a genuine smile and a false alarm for a polite smile identified as genuine. We computed d’ as a measure of participants’ ability to discriminate between the smiles. We estimated d’ with the following formula:

$$\text{d}’=Z_{H}–Z_{F}$$ (3)

where *Z*_*H*_ is the z-transformed probability of a hit and *Z*_*F*_ is the z-transformed probability of a false alarm. We also calculated a measure of the degree to which participants’ responses were biased toward one smile or the other (this measure is known as “criterion” in signal detection theory terms, [54]) and was calculated as:

$$\text{C}=-.5*\left(Z_{H}–Z_{F}\right)$$ (4)

with *Z*_*H*_ and *Z*_*F*_ as noted above. This allowed us to determine whether our manipulation altered participants’ ability to identify smiles, as previous research suggests [34], or introduced bias into their responses on our discrimination task. All post-hoc tests were Bonferroni corrected for multiple comparisons.

## Results/Discussion

**Fig 2:**
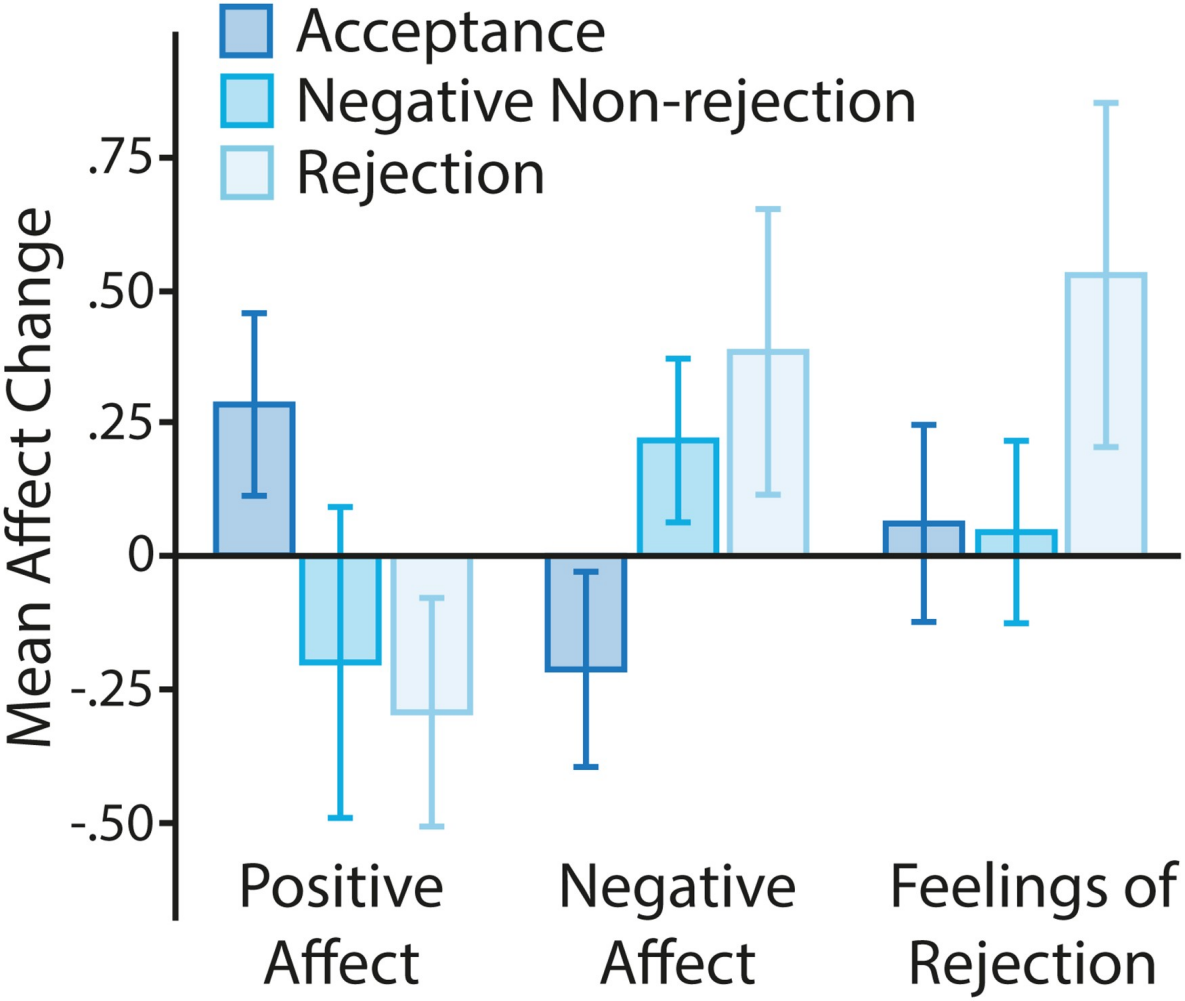
Affect by condition. Affect change (positive, negative and feelings of rejection) from pre- to post-manipulation. Error bars show the 95%CIs.

To ensure that the participants who received rejection feedback were the only participants who experienced changes in social state (feelings of rejection), we examined self-reported post-manipulation social state, controlling for pre-manipulation social state in an ANCOVA model (see Fig 2). The omnibus test showed significant changes from baseline feelings of rejection, F(2,86) = 5.557, p = .005, *η*^*2*^_*p*_ = .114. Post-hoc analyses showed that participants who received rejection feedback reported a significant increase in feelings of rejection relative to both acceptance (p = .029) and negative control conditions (p = .008). These data therefore suggest that only the rejection manipulation altered social state.

**Fig 3:**
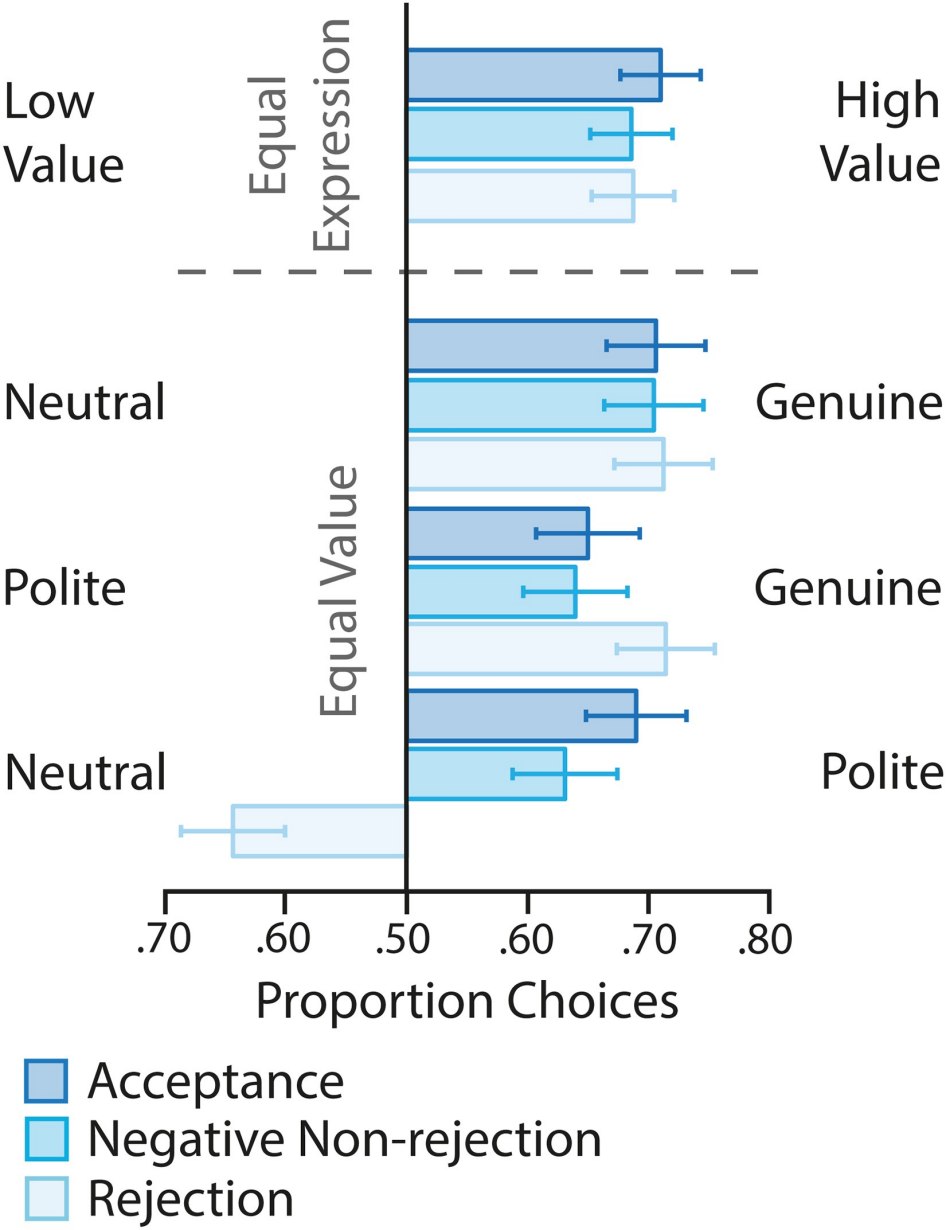
Choice behaviour. Average proportion choices allocated to stimuli in various pairings in the smile valuation task depending on value (holding expression constant) and expressive display (holding value constant) across feedback groups. Error bars show the 95%CIs.

**Fig 4:**
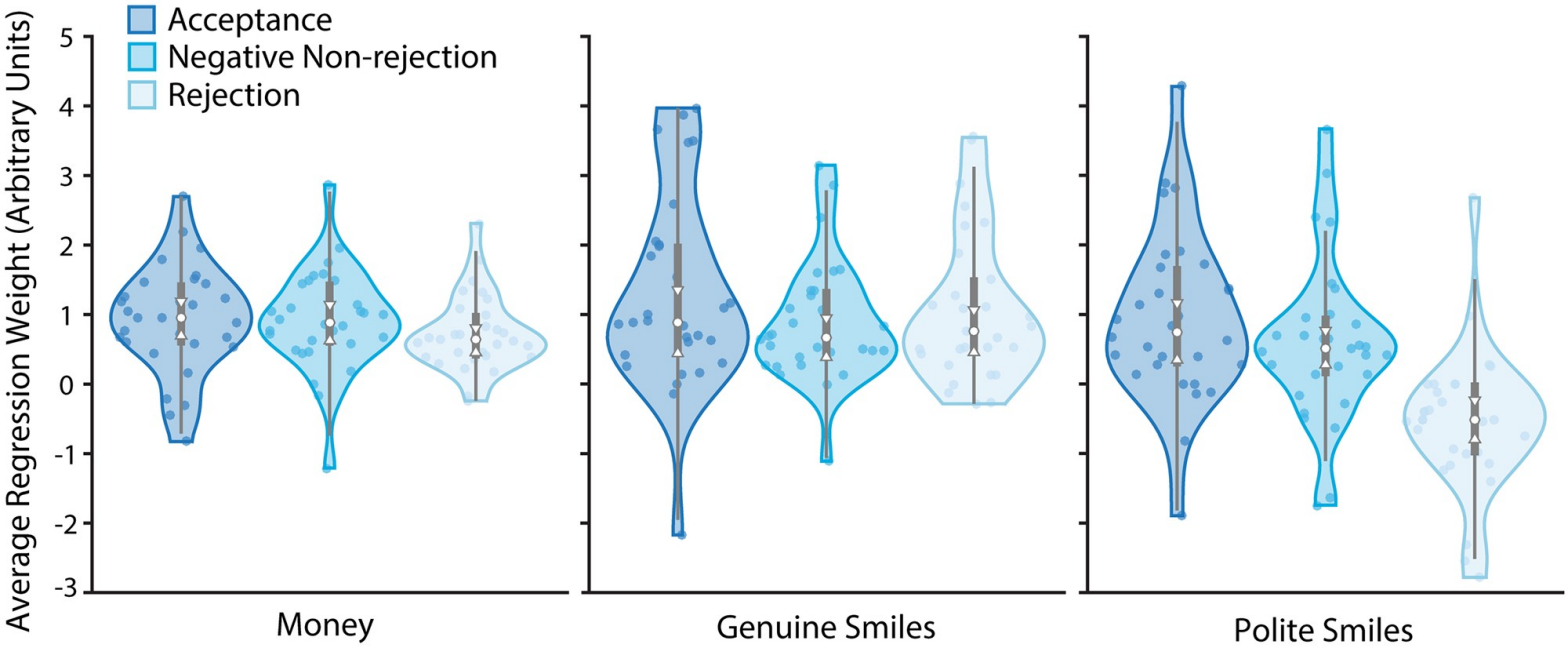
Regression analyses. Violin plots of regression weights estimated by the logistic model, indicating the degree to which money, genuine smiles, and polite smiles influenced choice behaviour across conditions. The grey-shaded central boxes show the inter-quartile range and the whiskers show the 95^th^ percentile of the data distribution. Plot upper and lower boundaries show the full range of the data. The white dots depict the distribution medians while the notches show the 95% confidence interval on the medians. Individual data points are marked with coloured dots on each plot.

Fig 3 shows the proportion of choices participants allocated to each face in a given pairing. A MANOVA model examining differences in the regression weights across conditions showed that there were no differences in the degree to which money influenced choice behaviour, regardless of the social feedback participants received, F(2,87) = 1.399, p = .252, *η*^2^_p_ = .031 (Fig 4). Based on the fact that the regression coefficients were significantly greater than zero (t(29)-values>5.074, p-values<.001), all participants treated genuine smiles as valuable stimuli. There were no group differences in how they treated these smiles, F(2,87) = .957, p = .388, *η*^2^_p_ = .022.

In contrast, the value of polite smiles was specifically influenced by the rejection feedback, F(2,87) = 14.563, p<.001, *η*^2^_p_ = .251 (Fig 4). Participants receiving acceptance and negative, non-rejection feedback treated polite smiles as positive stimuli (regression coefficients significantly greater than zero; t(29)-values>2.831, p-values<.008). These groups did not differ from one another (p = .715). For participants receiving rejection feedback polite smiles became aversive stimuli (regression coefficient smaller than zero; t(29) = -3.023, p = .005). This value differed significantly from both other social feedback groups (p-values<.001). Thus, social rejection influenced the value of polite, but not genuine smiles and, although polite smiles influenced other participants’ choices positively, polite smiles lost their value for participants in the heightened state of social need participants experienced following rejection feedback.

Comparing the regression weights for genuine and polite smiles with those for money allowed us to estimate the subjective value of a smile in monetary terms. The value of a genuine smile was 1.390 pence (95%CI = [.830, 1.950]), .972 pence (95%CI = [.616, 1.328]), or 1.617 pence (95%CI = [1.020, 2.214]) for the acceptance, negative-non-rejection and rejection groups respectively. Polite smiles were worth 1.026 pence (95%CI = [.536, 1.517]) and 0.661 pence (95%CI = [.183, 1.138]) for the acceptance and negative non-rejection groups. For rejected participants however, polite smiles were worth -.849 pence (95%CI = [-1.423, -.275]), meaning that the financial value of politely smiling faces would need to be about .85 pence higher than that of a neutral face to achieve an equal probability of the politely smiling face being chosen (point of indifference).

Finally, we examined perceptual sensitivity for genuine and polite smiles in a short smile discrimination task in which participants differentiated these smiles in order to determine whether potential differences in smile discrimination ability affected results. A MANOVA with d’ and criterion as the dependent variables and feedback type as the independent variable showed that there were no group differences in participants’ ability to differentiate genuine from polite smiles, F(2,87) = .340, p = .713, *η*^2^_p_ = .008 (Average d’: Acceptance = 1.856 [SD = 1.597]; Negative non-rejection = 1.608 [SD = .845]; Rejection = 1.592 [SD = 1.590]), nor did the groups differ in the degree to which they were biased to say a smile was genuine, F(2,87) = 0.623; p = .539, *η*^2^_p_ = .014 (Average criterion: Acceptance = .009 [SD = .603]; Negative non-rejection = -.099 [SD = .455]; Rejection = -.141 [SD = .548]). Thus, the differences in participants’ choice behaviour were not due to alterations in smile-discrimination ability.

Contrary to prediction, the main finding from Experiment 1 is that participants expecting to interact with a rejecting partner did not increase the degree to which they valued genuine smiles. Rather, genuine smiles held their value whereas polite smiles changed from positive to negative cues for these participants. Thus, although the difference between genuine and polite smiles increased dramatically for participants expecting rejection, this was entirely due to decreases in the valuation of polite smiles. A control experiment (see Supporting Information: Figures A and B in S1 File) showed that a simple positive/negative affect manipulation was not sufficient to alter decision-making in response to smiles. Thus, these data suggest that polite-smile-value is malleable and that polite smile devaluation appears to be specific to the altered social state our manipulation provoked. This finding suggests an important prediction for how changes in social state might alter nonverbal behaviour in face-to-face social environments. Specifically, participants who believe they are interacting with a potential rejecter should be less motivated/likely to respond to that person’s polite smiles, a prediction we now test.

## Experiment 2

If rejected participants do indeed devalue polite smiles, they should reciprocate a lower proportion of these smiles during a social interaction with a potential rejecter, relative to participants who do not expect rejection. Additionally, based on the fact that we did not see differences in genuine smile valuation in Experiment 1, we predict that genuine smile reciprocity should be unaffected by the manipulation.

## Methods

### Participants

We recruited 144 participants (mean age: 21.90, SD = 2.64) for a study of “personality characteristics and first impressions.” Participants were pseudo-randomly assigned to same-sex dyads with the constraint that the age gap between participants be 3 years or less. There were 36 male and 36 female dyads. The number of dyads recruited was determined a priori, based on the Experiment 1 effect size (effect size f = .587, *α* = .05, 1-*β* = .95). The sample-size calculation suggested 16 dyads per condition. However, we again oversampled to achieve 24 dyads per condition after exclusions. This was the maximum we were able to recruit given time and financial constraints (i.e., the ability to get the data coded without external funding for the project). Participants received 7.00GBP for taking part in the study. We excluded data from seven additional dyads in which the target participant did not believe the feedback (four dyads); the dyad members knew one another (one dyad); or technical difficulties interfered with video recording (two dyads). As in Experiment 1, all participants scored less than 60 on the Interaction Anxiousness Scale.

### Procedure

Upon arrival, an experimenter escorted participants to separate rooms in which they completed the same personality questionnaires and manipulation as in Experiment 1. In this case however, only one ‘target’ member of each dyad (randomly assigned) received active feedback. The other member received no feedback. This meant that these ‘neutral’ participants did not approach the interaction with any manipulated expectations about interaction outcomes. The inclusion of neutral participants allowed us to determine how a social partner might perceive behaviour changes on the part of target participants.

Experiment 2 used the same three feedback conditions as Experiment 1. The feedback types were pseudo-randomly assigned to dyads such that 24 dyads received each feedback condition and half the dyads per condition were male pairs. Because the computer controlled feedback assignment, the experimenter remained blind to both which participants received active/neutral feedback and which type of active feedback each dyad received.

Following the manipulation the experimenter escorted participants to an interaction room, introduced them, and left them alone with the instruction to converse for a few minutes on any topic. Conversations lasted 5 minutes and were captured on digital video. After the interaction, the experimenter returned participants to their individual rooms to complete a modified Desire for Future Interaction scale (DFI [55]) as a measure of partner liking. The DFI assesses the degree to which participants desire future meetings with the partner (e.g., “I would like to meet my partner again.”) using a 6-point Likert scale (1 = definitely no; 6 = definitely yes; present sample internal consistency: *α* = .84). Thereafter, the experimenter probed participants for suspicion, debriefed and paid them, explained the manipulation and allowed them to provide fully informed consent or have their data destroyed. No participant declined consent.

### Coders

Four research assistants independently coded participants’ smiles. All were naive to study hypotheses and participants’ feedback conditions. Before coding any data, coders were trained to 95% agreement on a set of pilot interactions. To check post-training reliability, each coder independently coded a series of three interactions, achieving 94% agreement. For the remaining interactions, two coders overlapped their coding for 50% of the sessions (36 sessions, both participants).

Coders viewed one participant at a time (video only; a computer program suppressed the audio files during coding) and coded that participant’s behaviour for the entire 5-minute interaction before coding another participant’s data. We recorded interactions at a rate of 25 frames-per-second and used frame counts to identify the onsets of each smile and to link dyad members’ behaviour. Each time they saw a smile, coders noted the frame at onset, frame at offset and type of smile produced. To differentiate the smiles, coders used a FACES-based coding system [56] and classified smiles as genuine if they involved the eye region and polite if they did not. Cohen’s kappa coefficients, which assess inter-rater agreement, showed excellent reliability for both smile types (genuine: .93, polite, .91, see [57]). Coder disagreements were resolved by consensus.

### Data analysis

To determine instances of smile reciprocity for each target participant, we used a purpose-written MATLAB script to examine the coded sessions for each smile that the neutral partner initiated (i.e., the target participant was not smiling and the neutral partner began smiling) and marked a reciprocated smile if 1) the target participant smiled back at the partner within four seconds (see [10]), and 2) the return smile was the same smile type as the initiating smile (genuine or polite). We used these data to calculate the proportion of partner smiles each participant returned for each smile type.

The design included four *participant types* (neutral, acceptance, rejection, negative non-rejection), nested within three *dyad types* (acceptance, rejection, negative non-rejection). Because we were focused specifically on the effects our social state manipulation had on the proportion of genuine and polite smiles participants returned, we excluded the neutral partners from the analysis of smile reciprocity. This effectively removed the source of the interdependence between participants nested within a given dyad (see [58, 59–61]). All post-hoc comparisons used Bonferroni’s correction for Type I error.

## Results/Discussion

**Fig 5:**
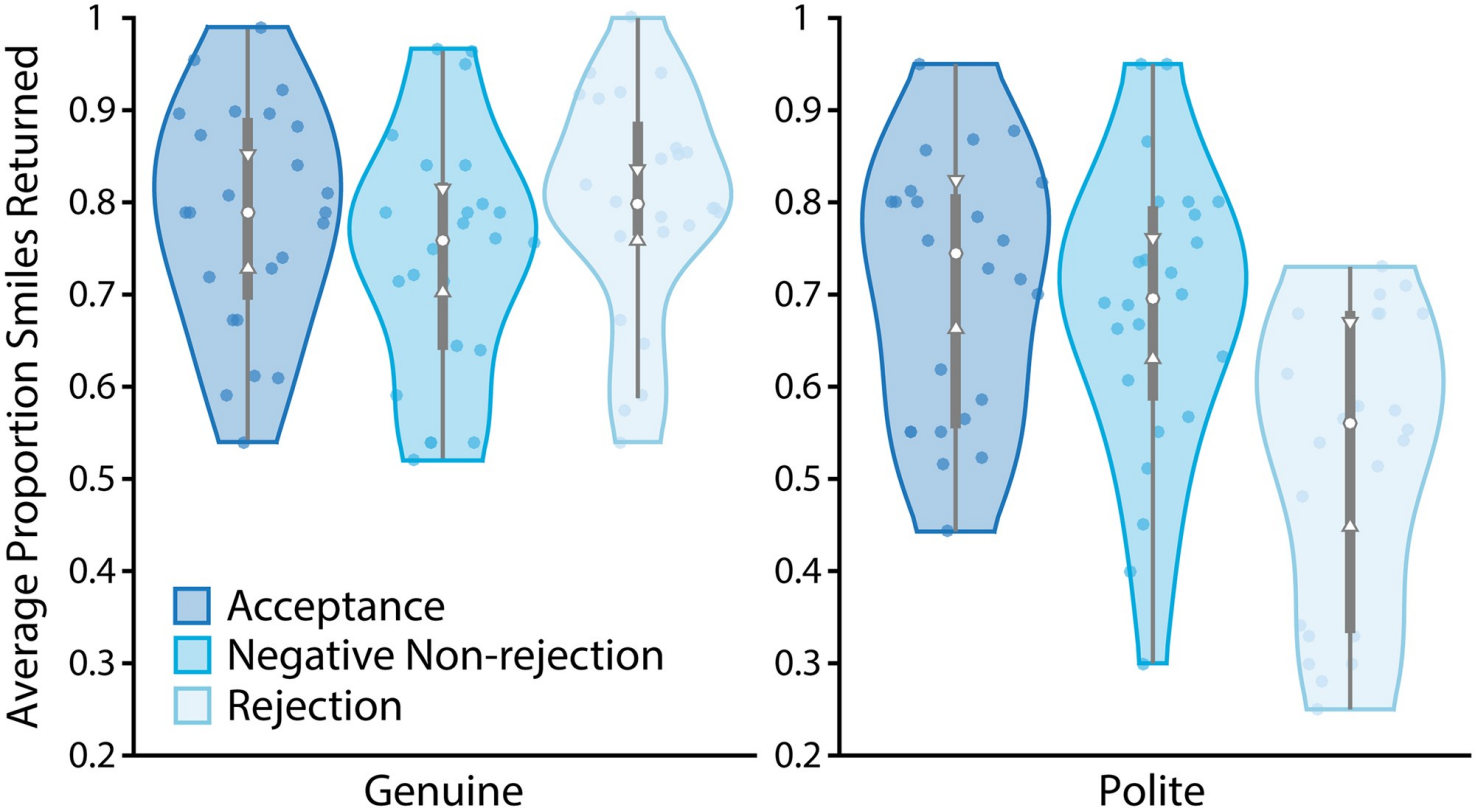
Smile reciprocity. Violin plots showing percent genuine and polite smiles returned in face-to-face interaction depending on participant condition. The grey-shaded central boxes show the inter-quartile range and the whiskers show the 95^th^ percentile of the data distribution. Plot upper and lower boundaries show the full range of the data. The white dots depict the distribution medians while the notches show the 95% confidence interval on the medians. Individual data points are marked with coloured dots on each plot.

As Fig 5 shows, target participants reciprocated similar proportions of their partners’ genuine smiles across feedback conditions, F(2,69) = 1.286, p = .283, *η*^2^_p_ = .036, meaning that as anticipated, the feedback did not affect genuine smile reciprocity across participant-types. However, when we examined the proportion of polite smiles target participants returned, the predicted effect was significant, F(2,69) = 10.136, p<.001, *η*^2^_p_ = .227. These results suggest that participants who interacted with a partner they believed had rejected them returned a significantly lower proportion of their partners’ polite smiles relative to those who believed that the partner liked them (p<.001) and those who received negative non-rejection feedback (p = .002). These data suggest that our social-state manipulation shaped real-world social behaviour in a manner consistent with the Experiment 1 findings. That is, participants reduced their polite smile reciprocity in an interaction with a potential rejecter, without altering genuine smile reciprocity. These results therefore serve as a conceptual replication of Experiment 1.

**Fig 6:**
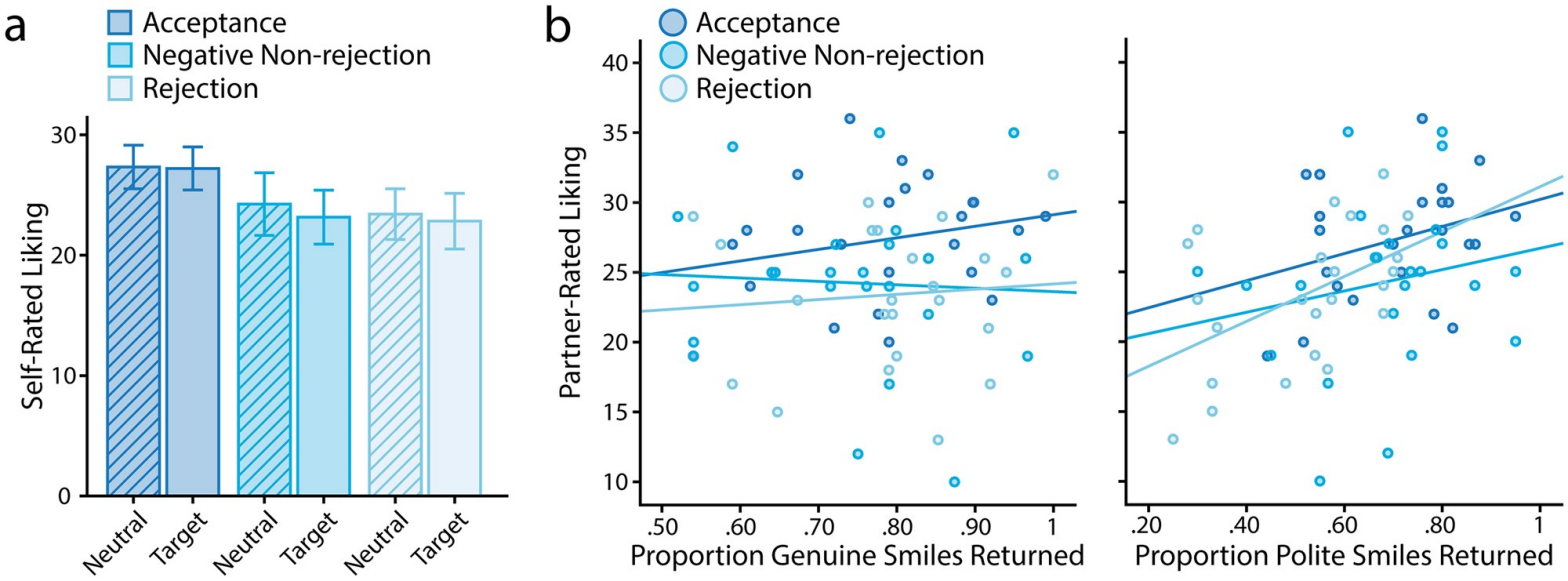
Liking ratings. **a)** Self-reported liking of the partner across participant conditions, including both neutral (hatched bars) and target participants within dyads. Error bars show the 95%CIs. **b)** Relationship between proportion of genuine and polite smiles participants returned and the partner’s liking rating.

One important question that this study methodology allows us to ask relates to how much neutral participants notice such seemingly subtle changes in behaviour. To investigate this question, we examined how much participants reported liking their partners. Unsurprisingly, target participants in acceptance dyads liked their partners better than did participants in other dyad types (Fig 6a; p-values<.021; Omnibus F(2,69) = 5.604, p = .006, *η*^2^_p_ = .140), who did not differ from one another (p>.999). Interestingly, neutral partners’ ratings of targets showed a similar pattern (Omnibus F(2,69) = 3.770, p = .028, *η*^2^_p_ = .099; acceptance > rejection: p = .034; no other comparisons were significant after Bonferroni correction: p-values>.132). To understand whether participants’ smile reciprocity predicted this effect, we selected only target participants and regressed their feedback-type, self-reported liking for the partner, and genuine and polite smile reciprocity on the partner’s liking for them in a hierarchical model (Table 1; Fig 6b). Results showed that after accounting for feedback-type and participants’ own liking ratings, polite smile reciprocity predicted the degree to which the neutral partner liked them. The effect of genuine smile reciprocity was not significant. These results suggest that as in previous research [62] this seemingly small change in social behaviour was both noticeable to neutral partners and has important social consequences related to the likelihood of future relationship development.

**Table 1:** Summary of hierarchical regression analysis of variables predicting partner liking ratings.

Variable	β	*t*	*p-value*	*R*	*Adj R*^*2*^	*ΔR*^*2*^
Step 1				.298	.076	.089
Feedback Type	-.298	-2.611	.011			
Step 2				.368	.110	.047
Feedback Type	-.221	-1.859	.067			
Liking for Partner	.229	1.930	.058			
Step 3				.479	.183	.094
Feedback Type	-.073	-.575	.567			
Liking for Partner	.254	2.219	.030			
Genuine Smile Reciprocity	.065	.597	.552			
Polite Smile R eciprocity	.326	2.703	.009			

Note: N = 72; Analysis includes target participants only. Step 1 FΔ (1,70) = 6.818, p = .011; Step 2 FΔ (1,69) = 3.726, p = .058; Step 3 FΔ (2,67) = 4.076, p = .021

## General discussion

Together, these results suggest that a social cue’s subjective value affects the moment-to-moment social decisions people make in response to that cue. Specifically, participants induced to feel states of high social need based on receiving information about possible rejection demonstrated significant polite smile devaluation and reduced reciprocity for polite smiles during naturalistic face-to-face interaction. The fact that complementary findings occurred in a computer-based economic task and in participants’ moment-to-moment social decisions is, to our knowledge, the first direct evidence that social-state dependent modulation of social-cue utility has real-world consequences. This finding suggests that reward-based decision-making may govern at least some of the moment-to-moment behaviours people produce in natural social environments.

How might such decision-making work? One suggestion from the literature is that people pay more attention to cues associated with high-value outcomes [63, 64]. Insofar as this is true, it might be the case that people are less likely to attend to low-value behaviours and consequently less likely to respond to those cues [10, 65]. However, because people generally experience predictable smile reciprocity from their partners [10, 37], reduced smile-return rates may be experienced as unpleasant and eventually cause discomfort and dislike.

One major implication of this set of findings is that neuroeconomic approaches to social stimuli may offer real opportunities for predicting how internal experiences such as emotions drive both the social behaviour people produce and their evaluations of partners’ behaviour. Specifically, neuroeconomic methods allow the quantification of relative stimulus utility more precisely than traditional psychological methods involving Likert rating scales. These methods therefore render the highly subjective social stimuli people encounter, such as smiles, tractable to prediction under formal mathematical models. This is a necessary step in understanding how the neural systems that underpin social decision-making function to control face-to-face social behaviour.

Importantly, the general affective changes associated with anticipating social rejection cannot explain our findings. Participants in the negative non-rejection condition, who did not experience alterations in social state, showed no changes in social decision-making although they reported similar affect changes as participants anticipating rejection. This implies that a specific social state manipulation is required to alter the utility of a polite smile. Data from our control study (Supporting Information: Figures A and B in S1 File) confirm this result, showing that a simple emotion induction is not sufficient to alter smile utility.

Our results cannot be due to social-state-dependent alterations in the ability to differentiate smiles because our manipulation did not alter participants’ ability to distinguish genuine from polite smiles, nor did it induce response bias. This finding runs contrary to previous findings (e.g., [34]). However, we note that our smile stimuli utilize a much more controlled set of still images, selected based on the degree to which they were discriminable, rather than the video smile-stimuli used in previous research.

As in much social psychological research, we have estimated social value in the relatively abstract setting of the laboratory, by measuring decisions to static face stimuli on a computer screen. However, unlike the majority of research in this field, we also show that these laboratory findings specifically predict behaviour in naturalistic social interactions. We think this combination of methodologies, in which we examine a prediction from the laboratory in the more complex world of face-to-face social interaction, is a critical step toward ensuring the ecological validity of psychological results.

Of course, the face-to-face interactions we report are limited by the fact that they took place only between target participants and people they believed had directly evaluated and possibly rejected them. Thus, our findings may not converge neatly with other rejection-related findings showing mimicry increases after rejection (e.g., [66]) because one would expect to see less prosocial behaviour during interactions with rejecters [21, 49] compared to neutral or inclusive individuals as in [66]. We also examined only one type of social state manipulation–social rejection. It is therefore important that future research examine the influence of different social-state manipulations on both cue valuation and social behaviour. Nonetheless, our findings suggest that cue-value models provide leverage for understanding how typical participants make behavioural decisions in face-to-face interactions.

In summary, we demonstrate evidence of social-state-dependent fluctuations in the degree to which people subjectively value social rewards. Our findings show that both genuine and polite smiles are rewarding, relative to neutral faces. However, in states of high social need, polite smiles turn from mildly positive into negative social stimuli. This effect translates to real-world social decisions by reducing the frequency with which those anticipating social rejection respond to a social partner’s polite smiles. Thus, the degree to which a social cue influences social decisions depends profoundly on the receiver’s social state and suggests that understanding how people subjectively value the social stimuli they receive may provide important evidence for understanding how the brain controls and tunes the moment-to-moment production of social behaviour.

## Supporting information

S1 Fig False feedback pilot data. Pilot participants’ perceptions of acceptance, rejection and negative non-rejection feedback. Error bars show the 95%CI. P-values for feedback-type comparisons are Bonferroni corrected.
(PDF)

S1 File Non-social affect induction control experiment. Methods and results of a control experiment examining whether a simple positive and negative affect induction caused changes in polite smile valuation.
(PDF)
