# Supplementary material for: The subjective value of a smile alters social behaviour
Source: PLoS One. 2019 Dec 2;14(12):e0225284. doi: 10.1371/journal.pone.0225284 (PMC6886806; doi:10.1371/journal.pone.0225284)
Supplement: S1 Fig — Pilot participants’ perceptions of acceptance, rejection and negative non-rejection feedback. Error bars show the 95%CI. P-values for feedback-type comparisons are Bonferroni corrected. (PDF) [file pone.0225284.s001.pdf]

False Feedback Pilot Results

In order to ensure that the false feedback was believable, participants completed a 7-item inventory in which they rated how much they looked forward to the interaction. The inventory contained the following items, rated on a 5-point Likert scale (1=Not at all; 5=Extremely). We note that in the datasets reported in the main text, prior to receiving the false feedback, there were no group differences on this measure (Experiment 1 sample: $F(2,87)=.752$, $p=.457$; Experiment 2 sample: $F(3,140)=.460$, $p=.711$).

- I think I will enjoy meeting my partner.
- I'm feeling sociable today.
- I'm looking forward to the interaction.
- I'm likely to have something in common with my partner.
- I'm not likely to enjoy interacting with my partner. (Reversed)
- My partner does not seem like the sort of person I usually get along with. (Reversed)
- I have personal reasons (e.g., stressed over work, school) for not looking forward to the interaction. (Reversed)

Using these items, we created several versions of each of three false feedback conditions. The exact wording in each condition was chosen based on a small pilot sample's rating of the degree to which the feedback was believable, as well as how pilot participants reported that they would feel encountering each version of the feedback. All versions of each condition had the desired effect in terms of participants' affective experience. We therefore chose the feedback version that participants rated as most believable in each condition. The exact wording of each feedback condition was: Acceptance ("Your partner is feeling sociable. Your partner thinks there is a good chance that the two of you will get along. Your partner is looking forward to meeting you."); Rejection ("Your partner does not think the two of you will have anything in common. Your partner does not think the two of you will get along. Your partner is not looking forward to meeting you."); and Negative non-rejection; ("Your partner is not feeling very sociable. Your partner has personal reasons for not looking forward to the interaction. Your partner is not looking forward to meeting you.").

To ensure that the feedback generated the appropriate affect changes along with consistent judgments of the partner from whom it ostensibly came, an independent sample of 32 participants read each of the finalized false feedback statements and rated it for the degree to which they believed it would generate positive affect, negative affect, and feelings of rejection. They also rated the items based on the attributions the feedback would cause them to make (internal to myself; internal to the partner; external factors) and whether they would feel empathy for the partner who provided it. All items were posed as statements (e.g., "If I received this feedback, I would feel more positive than before receiving the feedback") and rated on a 4-point scale (1=Strongly agree; 4=Strongly disagree).

Repeated measures ANOVAs generated significant omnibus tests indicating feedback-type differences for affect (positive, negative, feelings of rejection) and for internal-self and internal-partner attributions (all p-values<.001) and empathy ($p=.010$) but not external attributions ($p=.572$). Fig S1 shows feedback-related differences in the ratings (and Bonferroni-corrected p-values for all post-hoc comparisons). Participants agreed that acceptance feedback would

promote greater positive affect than either rejection or negative non-rejection feedback. They also agreed that rejection and negative non-rejection feedback would promote negative affect and acceptance feedback would not. Importantly, participants agreed that only rejection feedback would promote feelings of rejection. With respect to attributions about the causes of the feedback, participants agreed that they would believe acceptance and rejection feedback were due to stable internal aspects of the self (but not the partner) and that the reverse was true of the negative non-rejection feedback. Participants disagreed that they would attribute any feedback type to external factors. Finally, participants tended to either disagree or strongly disagree that they would experience empathy for the partner. Together with the affect data reported in the main text, these data suggest that the false feedback achieved its desired effect.

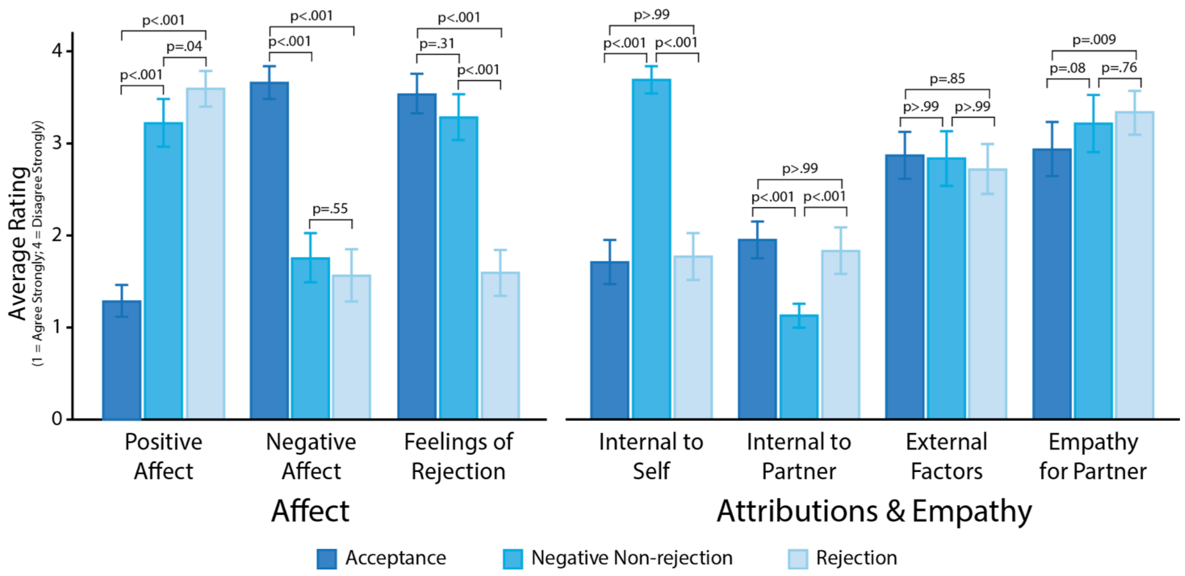

Fig S1. False feedback pilot data. Pilot participants' perceptions of acceptance, rejection and negative non-rejection feedback. Error bars show the 95%CI. P-values for feedback-type comparisons are Bonferroni corrected.
