# Supplementary material for: The subjective value of a smile alters social behaviour
Source: PLoS One. 2019 Dec 2;14(12):e0225284. doi: 10.1371/journal.pone.0225284 (PMC6886806; doi:10.1371/journal.pone.0225284)
Supplement: S1 File — Methods and results of a control experiment examining whether a simple positive and negative affect induction caused changes in polite smile valuation. (PDF) [file pone.0225284.s002.pdf]

Non-social Affect Induction: Control Experiment

To ensure that social state, rather than emotional state more generally was the important predictor of alterations in smile utility, we conducted a version of Experiment 1 using a standard affect manipulation, designed to induce either positive or negative mood states (Gerrards-Hesse, Spies, & Hesse, 1994). In this experiment, participants (N=68; 54 females) did not expect a social interaction. Instead, they began the session, with the same mood questionnaire used in Experiment 1. Mood induction occurred using a reaction-time task with manipulated difficulty. Each trial of the task began with a blank, black screen for 100ms, followed by the cue (a centrally presented grey asterisk), which remained visible for 100ms. Participants pressed the space bar as quickly as possible after the cue appeared. Between trials, the computer randomly selected an inter-stimulus interval (ISI) from a normal distribution with a mean of 1200ms. The screen remained blank for the duration of the ISI before the start of the next trial. The blank screen served as both the response window and the ISI. The computer did not wait for responses before presenting the next trial. Participants were told that a response would be considered correct if they responded within 350ms of the cue's appearance.

The computer randomly assigned approximately half the participants (n=33) to receive a positive mood induction and the remainder to a negative mood induction (n=35) in a double-blind fashion. To induce positive affect, the computer made the task easy by adjusting the standard deviation of the ISI distribution so that stimuli were more predictable. In this condition, the ISI distribution standard deviation began at 40ms and, after the first 20 trials, adjusted to ensure accuracy scores of ~90%. At the end of each block of 50 trials, participants received praise (e.g., "Well done! You have detected [91]% of the targets within the response window. Keep up the excellent work.").

The negative mood induction altered the standard deviation of the ISI distribution to make the stimulus onset less predictable and the task more difficult. The ISI distribution standard deviation started at 400ms and adjusted (after the first 20 trials) to hold performance at ~60% correct. Participants received negative feedback from the computer at the end of each block (e.g., "Please try to concentrate! You have detected only [59]% of the targets within the response window. Please do your best to improve your detection rate."). Participants completed five blocks of 90 trials each. After the mood induction, participants repeated the mood inventory to measure change from baseline and then completed the same smile valuation task as in Experiment 1.

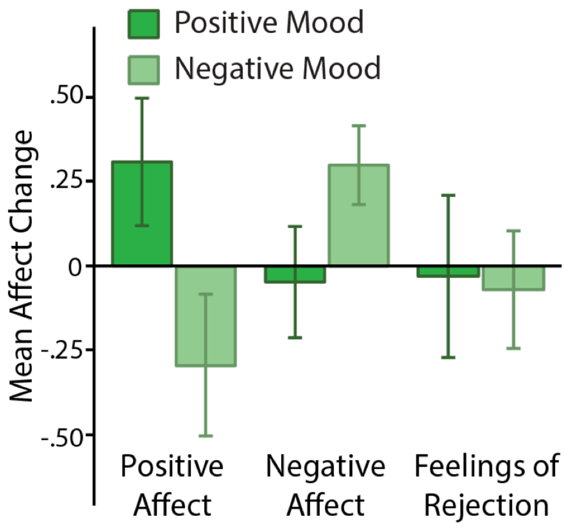

Fig A. Affect change. Affect change from pre- to post-manipulation for positive affect, negative affect and feelings of rejection after a positive or negative mood induction. Error bars show the 95%CI.

To ensure that the manipulation worked as expected, we compared participants' mood change scores for positive affect, negative affect and feelings of interpersonal rejection. As Fig A shows, participants receiving the positive mood induction felt significantly more positively, $F(1,63)=15.711$; $p<.001$; $\eta^2=.200$, and less

negatively than did those receiving the negative mood induction, $F(1,63)=13.468$; $p=.001$; $\eta^2=.176$. There were no differences in feelings of rejection, $F(1,63)=.582$; $p=.448$; $\eta^2=.009$, nor did either manipulation cause feelings of rejection to change from baseline levels (p-values>.412).

Based on the choice data (Fig Ba), we estimated the degree to which money and both smile types (relative to neutral faces) contributed to choice behaviour using the logistic model described in Experiment 1. There were no differences between the positive and negative affect groups in terms of how money, $F(1,66)=.046$; $p=.831$; $\eta^2=0.001$, genuine smiles, $F(1,66)=.133$; $p=.716$; $\eta^2=.002$, or polite smiles shaped participants' decisions, $F(1,66)=.006$; $p=.939$; $\eta^2<.001$ (Fig Bb). For participants in the positive affect condition, the value of a genuine smile was 1.387 pence (95%CI=.535; 2.238) and polite smiles were worth .967 pence (95%CI=.146; 1.789). For those in the negative condition, genuine smiles were worth 1.477 pence (CI=.756; 2.199) and polite smiles still carried a positive value of .933 pence (95%CI=.332; 1.535), contrasting with the negative value after the social rejection manipulation in Experiment 1. Together, these data show similar values for genuine and polite smiles as the Experiment 1 participants who experienced the acceptance and negative non-rejection conditions. Thus, these data suggest that social, rather than simple affective state changes are necessary to alter smile value.

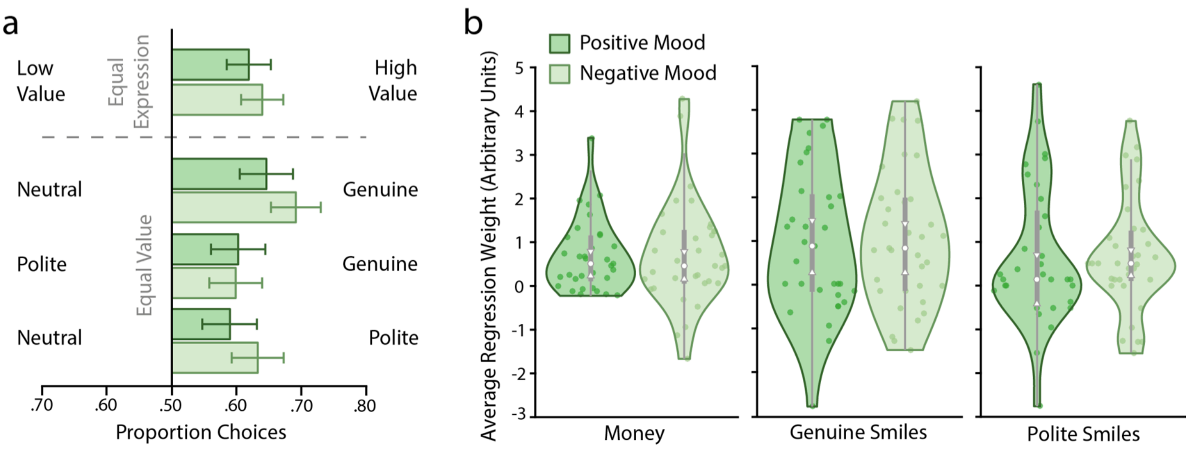

Fig B. Choice behaviour in the smile valuation task. a) Average proportion of choices allocated to stimuli depending on value (holding expression constant) and expressive display (holding value constant) across mood induction groups. **b)** Unstandardized logistic regression weights. Results indicate the degree to which money, genuine smiles and polite smiles influence choice behaviour across positive and negative mood groups. The grey-shaded central boxes show the inter-quartile range and the whiskers show the 95th percentile of the data distribution. The upper and lower boundaries of each plot show the full range of the data. The white dots depict the distribution medians while the notches show the 95% confidence interval on the medians. Individual data points are marked with coloured dots on each plot.

Reference:

Gerrards-Hesse, A., Spies, K., & Hesse, F. W. (1994). Experimental Induction of Emotional States and Their Effectiveness. A Review. British Journal of Psychology, 85, 55-78.
http://dx.doi.org/10.1111/j.2044-8295.1994.tb02508.x
